# Dust-concentration measurement based on Mie scattering of a laser beam

**DOI:** 10.1371/journal.pone.0181575

**Published:** 2017-08-02

**Authors:** Xiaoyu Yu, Yunbo Shi, Tian Wang, Xu Sun

**Affiliations:** The Higher Educational Key Laboratory for Measuring & Control Technology and Instrumentations of Heilongjiang Province, School of Measurement–Control Technology and Communications Engineering, Harbin University of Science and Technology, Harbin, China; Ludwig-Maximilians-Universitat Munchen, GERMANY

## Abstract

To realize automatic measurement of the concentration of dust particles in the air, a theory for dust concentration measurement was developed, and a system was designed to implement the dust concentration measurement method based on laser scattering. In the study, the principle of dust concentration detection using laser scattering is studied, and the detection basis of Mie scattering theory is determined. Through simulation, the influence of the incident laser wavelength, dust particle diameter, and refractive index of dust particles on the scattered light intensity distribution are obtained for determining the scattered light intensity curves of single suspended dust particles under different characteristic parameters. A genetic algorithm was used to study the inverse particle size distribution, and the reliability of the measurement system design is proven theoretically. The dust concentration detection system, which includes a laser system, computer circuitry, air flow system, and control system, was then implemented according to the parameters obtained from the theoretical analysis. The performance of the designed system was evaluated. Experimental results show that the system performance was stable and reliable, resulting in high-precision automatic dust concentration measurement with strong anti-interference ability.

## Introduction

With the rapid development of China's economy, the rapid expansion of cities, and the accelerated process of urbanization, air pollution is becoming increasingly serious, and the deterioration of visibility caused by aerosol particles has increased. In recent years, continuous fog and haze have become common environmental pollutants in the Beijing and Tianjin areas. Haze affects people's physical and mental health. Moreover, solar radiation is reduced, causing a reduction in crop production. This haze also causes traffic accidents [1–4]. Dust (a suspension in which the particle diameter is not greater than 75 μm) is an important source of pollution that has seriously polluted the atmospheric environment and impacts human health. According to the National Standard of Environmental Air Quality Index, the designation PM10 describes the particulate matter in which the particle size less than or equal to 10 μm, and PM2.5 refers to fine particulate matter in which the particle size is less than or equal to 2.5 μm. In urban areas of China, PM2.5 and PM10 accounted for the largest proportion of air pollution, showing that the main factor affecting the air quality of large cities in China is particulate matter. Therefore, accurate monitoring and control of atmospheric airborne particles has become an urgent scientific research area.

At present, there are three types of PM2.5 determination methods that have been widely adopted by the environmental protection departments of various countries: gravimetry, the β-ray absorption method, and the oscillating microbalance. In 2011, China's Ministry of Environmental Protection implemented new standards, namely HJ618-2011, that specified the use of the weight method for determining PM2.5 in the environment. In the weight method, PM2.5 in the ambient air is trapped in a filter membrane of known mass using constant velocity extraction of quantitative volumetric air, and the concentration of PM2.5 is calculated according to the quality difference and volume of the sample before and after sampling.

The oscillating microbalance method relies on the change in oscillation frequency due to the mass change of the filter membrane. The mass concentration of particulate matter in the time interval was calculated according to the flow rate, the temperature of the field environment, and the air pressure.

The β-ray method is based on the absorption intensity of the β ray released by the particulate matter to carbon-14. After the particles are adsorbed on the filter paper tape, a Geiger counter measures the concentration of the adsorbed particulate matter by measuring the variation of the β-ray intensity before sampling.

The light scattering method for measuring mass concentration is based on the Mie scattering theory of particles. When light strikes suspended particles in the air, the light scatters. For certain particle properties, the intensity of the light scattered off the particle is proportional to its mass concentration. By measuring the intensity of the scattered light, the particle mass concentration can be obtained by applying the conversion coefficient. Light passes through the particle matter under certain conditions based on the quantity of particles and the particle size approximately equal to the wavelength of the light.

China promulgated “ambient air quality standards” that limited the value of PM2.5 in 2012, and in large and medium cities, these standards were used to gradually establish and improve the PM2.5 monitoring network. However, even though there is a growing concern about the dangers of indoor PM2.5 levels on human health, at present, there is a lack of regulatory policies and control measures for PM2.5.

The monitoring of dust particle concentration has become increasingly important, and on-line rapid detection instrumentation is the primary developmental trend in dust particle concentration detection.

## Materials and methods

### Principle of measurement

According to Mie scattering theory, light scattering occurs when the beam illuminates an inhomogeneous medium, and a particle size approximately equal to the wavelength of the light. The relative scattering intensity varies as a function of angle. Based on the scattering theory of light and the Mie scattering theory of dust particles, the exact solution for the scattering of a pair of particles can be obtained after dust concentration detection by strict mathematical deduction via Maxwell’s equations.

When the incident light passes through dust particles, the light scattering occurs. The dust particle concentration(*M*_*v*_) and the intensity of the scattered light should satisfy the relation [5]:

Mν=4π3γ2ρ(II0)3λ2νS∑i[I1(α,m,θ)+I2(α,m,θ)nγ(Di)]Di3ΔDi (1)

Here, *γ* is the distance between the particles and the light intensity detector; *ρ* is the relative density of suspended particles; *I* is the intensity of incident scattering light; *I*_*0*_ is the incident light intensity; *λ* is the wavelength of the incident light; ν is the direction of gas flow; *S* is the cross-sectional area of the tested gas flows in the pipeline. Moreover, *I*_*1*_ and *I*_*2*_ are the intensities of the vertically and horizontally polarized components of the scattered light, respectively, which are related to the idealized spheroid particle size *α*, refractive index *m*, and alpha particle scattering angle *θ*. Finally, *nr(D*_*i*_*)* is the mass size distribution normalized function of suspended particles, and *D* is the particle diameter, *n* refers to the number size distribution of the particles.

As shown in Eq (1), the mass concentration of dust particles should have a linear relationship with the scattered light intensity. Thus, if the wavelength of the incident light is known and an appropriate scattering angle is selected, the mass concentration of dust particles can be obtained through the scattered light intensity calculation.

#### Scattering intensity distribution characteristics of single particle

With the incident light and scattered light determining the scattering plane, the scattering intensity perpendicular to the in-plane component can be determined using the scattering amplitude function [6–8]:

$$I=\left(\frac{\lambda^{2}}{8\pi^{2}\gamma^{2}}\right)\left(i_{1}+i_{2}\right)I_{0}$$ (2)

$$I_{1}\left(\alpha ,m,\theta \right)={\left|S_{1}\right|}^{2}$$ (3)

$$I_{2}\left(\alpha ,m,\theta \right)={\left|S_{2}\right|}^{2}$$ (4)

The scattering amplitude functions *S*_*1*_ and *S*_*2*_ are given by:

$$S_{1}=\Sigma_{n=1}^{\infty }\frac{2n+1}{n\left(n+1\right)}\left[a_{n}\pi_{n}\left(\text{cos}\theta \right)+b_{n}\tau_{n}\left(\text{cos}\theta \right)\right]$$ (5)

$$S_{2}=\Sigma_{n=1}^{\infty }\frac{2n+1}{n\left(n+1\right)}\left[a_{n}\tau_{n}\left(\text{cos}\theta \right)+b_{n}\pi_{n}\left(\text{cos}\theta \right)\right]$$ (6)

Here, the parameters *a*_*n*_ and *b*_*n*_ can be defined as:

$$a_{n}=\frac{\psi_{n}\left(x\right)\psi_{n}\left(mx\right)-m\psi_{n}\left(mx\right)\psi_{n}\left(x\right)}{\xi \left(x\right)\psi_{n}\left(mx\right)-m\psi_{n}\left(mx\right)\xi_{n}\left(x\right)}$$ (7)

$$b_{n}=\frac{m\psi_{n}\left(x\right)\psi_{n}\left(mx\right)-\psi_{n}\left(mx\right)\psi_{n}\left(x\right)}{m\xi \left(x\right)\psi_{n}\left(mx\right)-\psi_{n}\left(mx\right)\xi_{n}\left(x\right)}$$ (8)

$$\pi_{n}\left(\text{cos}\theta \right)=\frac{p_{n}^{\left(1\right)}\left(\text{cos}\theta \right)}{\text{sin}\theta }$$ (9)

$$\tau_{n}\left(\text{cos}\theta \right)=\frac{dp_{n}^{\left(1\right)}\left(\text{cos}\theta \right)}{\text{d}\theta }$$ (10)

In the formulas, *α*_*n*_ and *b*_*n*_ are Mie coefficients, and *x = πα/λ*, which is a function that relates the first class half-integer-order Bessel function *J*_*n+1/2*_*(z)* and the second half-integer-order Hankel function *H*_*n+1/2*_*(z)*. Here, *π*_*n*_*(cosθ)* and *τ*_*n*_*(cosθ)* are Legendre polynomials that are related only to the scattering angle.

From the formulas (2)–(10), the scattering light intensity distribution of particles is related to three parameters, namely the particle refractive index *m*, the length of the incident light wave *λ*, and the particle size *α*. In the study, we select these three parameters for theoretical numerical simulations to understand that how the scattering light intensity is affected.

#### Influence of particle refractive index on scattering light intensity

The refractive index of the suspended particles is an important factor influencing the scattering light intensity. The refractive index is expressed as follows:

$$m=m_{1}+im_{2}$$

**Fig 1:**
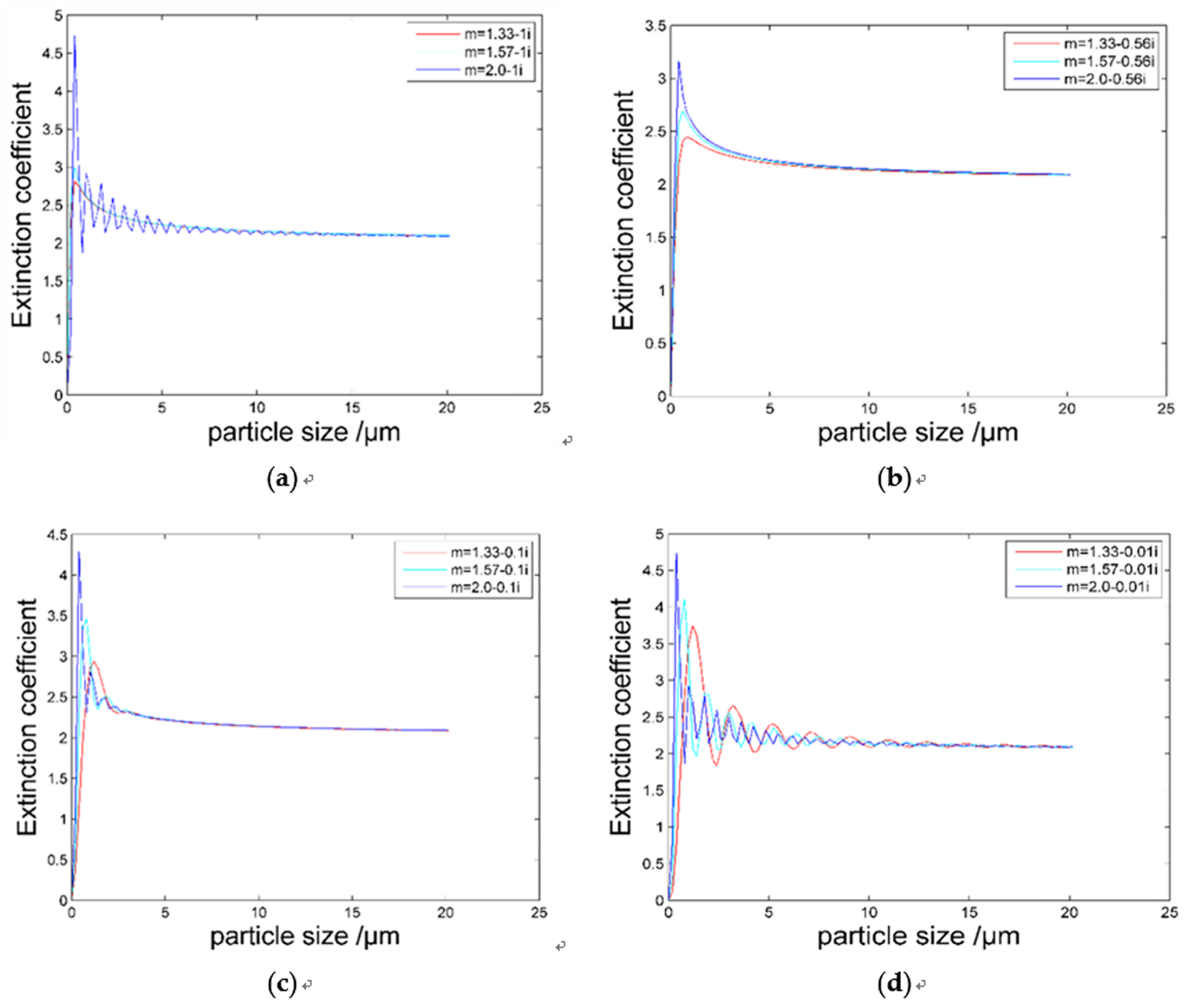
The relationship between particle size and net absorption for various values of the complex refractive index.

Here, *m*_*1*_ refers to the ratio between propagation velocity of light in vacuum and the propagation velocity in the medium. This numerical value is related to the properties of the material embodied as the scattering effect of incident light in the medium. On the other hand, *m*_*2*_ is the net absorption, which indicates the degree of the light intensity attenuation in the medium and describes the performance as the absorption effect of the medium to the incident light. The relationship between particle size and the relationship between particle size and net absorption for various values of the complex refractive index is shown in Fig 1, where the wavelength of the incident light is 650 nm.

In Fig 1(a), 1(b), 1(c) and 1(d), the real part of the refractive index is assumed to be 1.33 (the refractive index of water), 1.57 (the refractive index of cigarette ash), and 2.0 (the refractive index of dust in air), and the imaginary part, i.e., the extinction coefficient, is assumed to be 0.01, 0.1, 0.56, and 1, respectively [9,10]. From Fig 1, when the refractive index of the particles is not the same, the extinction coefficient of the same particle size is obviously different because the extinction coefficient curve significantly fluctuates. However, when the particle diameter is greater than 5 μm, the extinction efficiency gradually approaches a constant level of 2.

**Fig 2:**
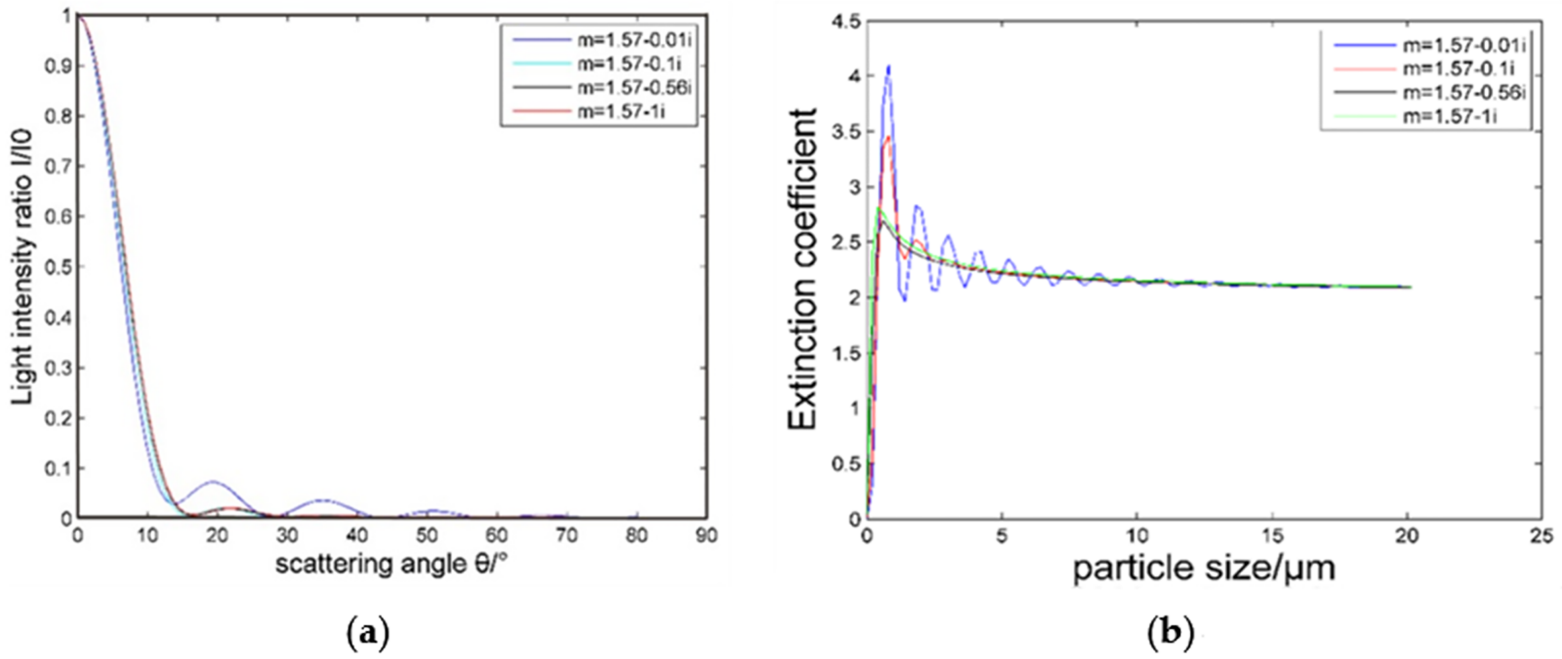
(a) The relationship between the imaginary part of the refractive index and the scattered light intensity distribution. (b) The relationship between the imaginary part of the refractive index and the dependence of the net absorption on particle size.

Fig 2 is a diagram that when the particle refractive index is not fixed, the relationship between the extinction efficiency and particle size when the imaginary part is not the same.

Fig 2(a) is the scattering light intensity distribution curve. The real part of the refractive index is 1.57, and the imaginary part is 0.01, 0.1, 0.56, or 1. At 20° scattering angle, the scattering intensity distribution is a very pronounced difference between the blue curve and the red curve, and there are slight differences in the intensity distribution of the scattered light between 20–60°. In Fig 2(b), below 5 μm, the blue curve is very different from the others.

From Figs 1 and 2, in the dust particle concentration measurement, when the real part is 1.57 and imaginary parts is 0.01 of the refractive index of the measured substance is significantly different, and the numerical effect on the scattering intensity is also different. Therefore, in the measurement, experimental error caused by the refractive index can be neglected.

#### Effect of incident light wavelength on the intensity of scattering light

**Fig 3:**
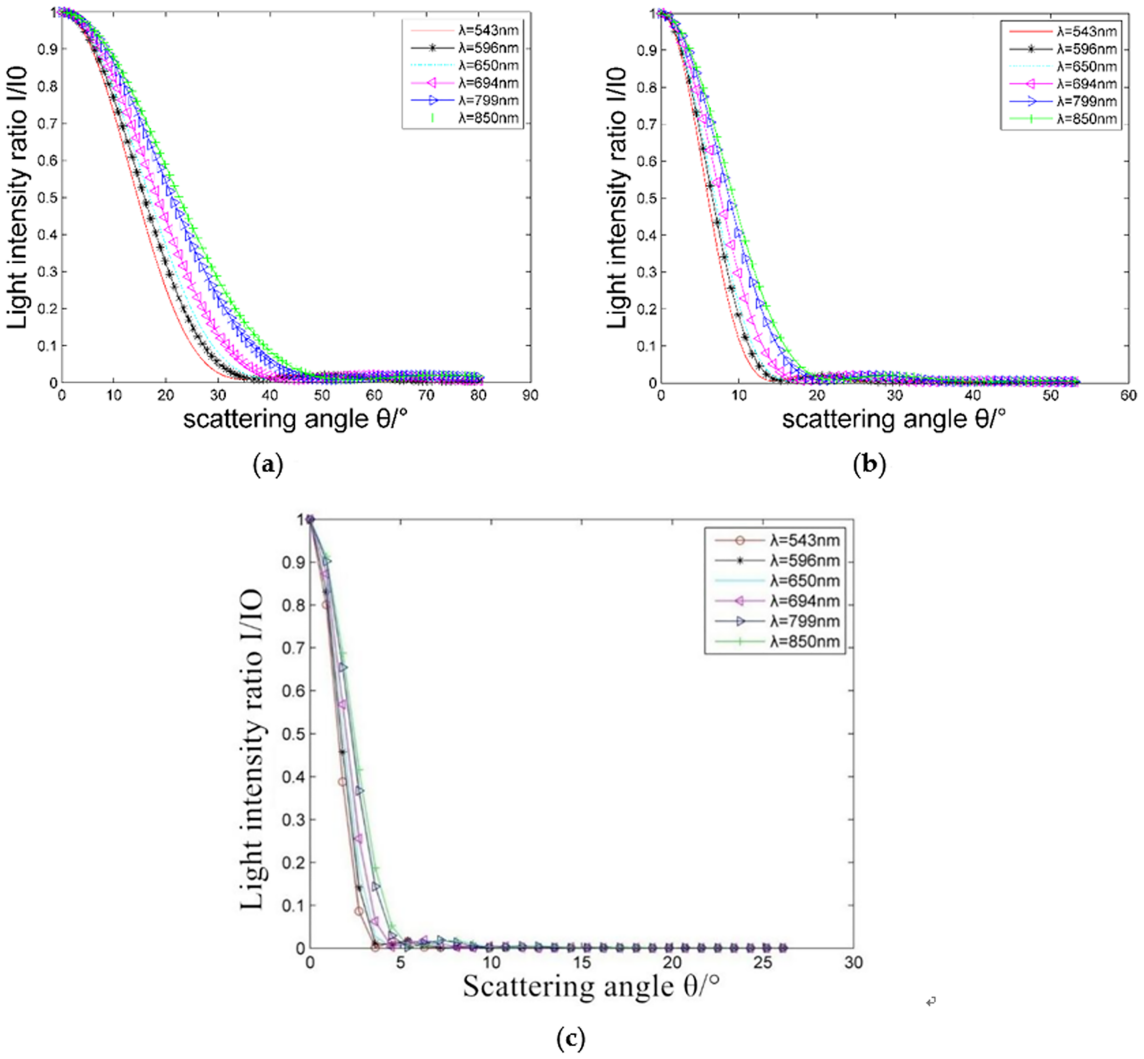
Scattering angular distribution curve of the scattered light intensity at different wavelengths.

Fig 3 shows the relationship between the scattered light intensity and the scattering angle at different incident light wavelengths when the refractive index of the particle is *m* = 1.57–0.56*i* and the particle sizes are 1 μm, 2.5 μm and 10 μm [Fig 3(a), 3(b) and 3(c), respectively].

From Fig 3(a), when the particle size is 1 μm, as the incident light wavelength increases, the scattering light intensity is primarily concentrated at small angles and gradually changes at slightly larger angles. As the wavelength of the incident light continues to increase, the scattering light intensity distribution also increases. When the particle diameter is 2.5 μm or 10 μm, the scattered light intensity distribution is more concentrated. Ideally, scattered light from the complete angular range of 0 to ±180° would have to be detected and measured to completely characterize the scattering properties of the particle. Since this goal is impractical, collecting light from the concentrated distribution is the next-best alternative. When the wavelength is less than 600 nm, the scattering light intensity of suspended particles is mainly concentrated within the scattering angle of 30°. When the wavelength is greater than 700 nm, the scattered light intensity of the suspended particles with small particle sizes is mainly concentrated within the scattering angle of 50°. The scattering angle of suspended particles is mainly concentrated within the 20°. Therefore, in the design of the system, a symmetrical arrangement of two photoelectric sensors is required to receive as much scattered light as possible.

### Effect of particle size of suspended particles on the intensity of scattered light

**Fig 4:**
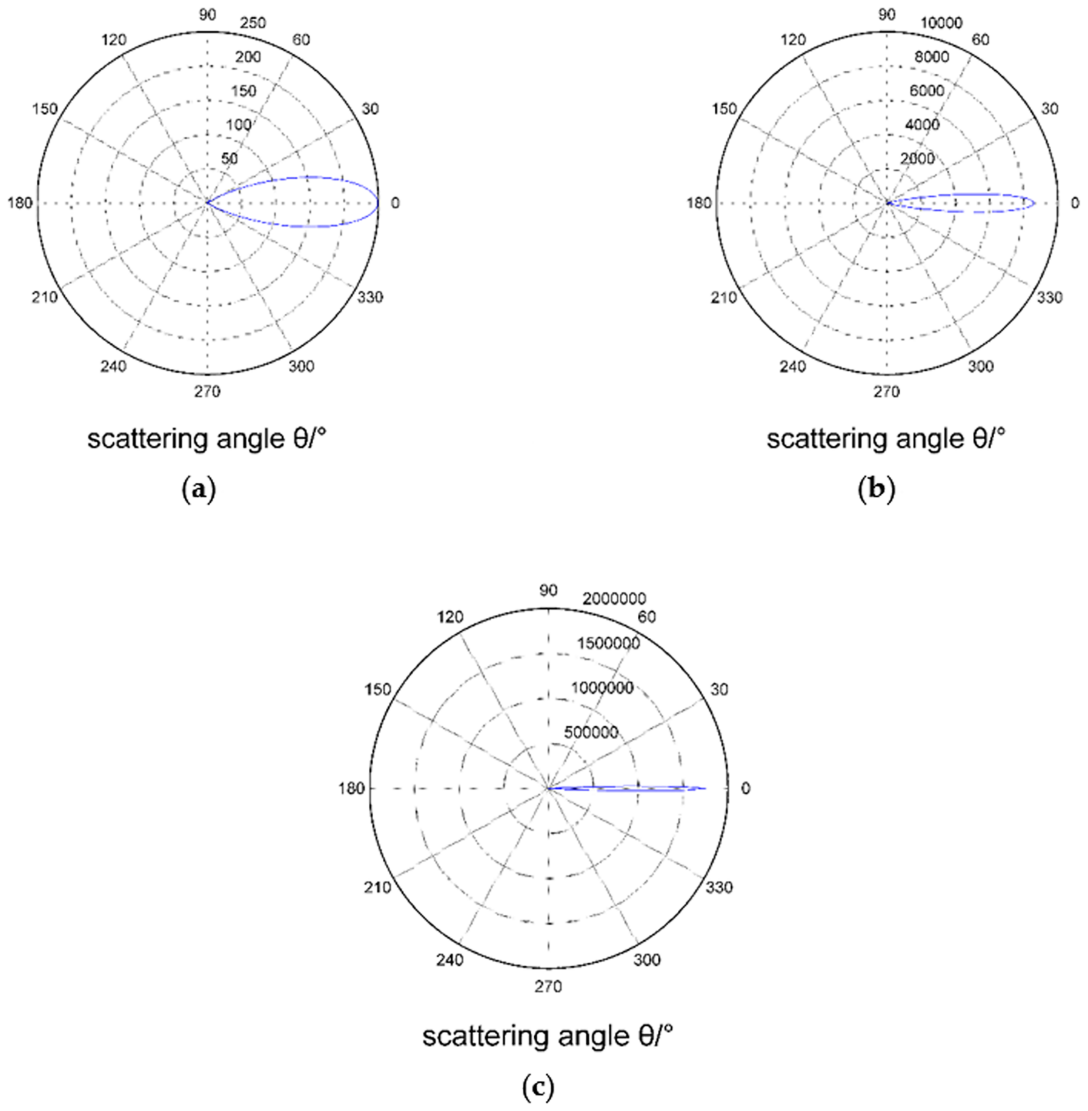
(a) and (b) show scattering angular distribution curves of vertical and horizontal components respectivelyof scattering light intensity of different particles for *m* = 1.57–0.56*i*, λ = 632.8 nm, and a particle diameter of 10 μm.

Fig 4 shows the relationship between the vertical and horizontal components of the scattering light intensity of different particles and the scattering angles, where the refractive index of particles is *m* = 1.57–0.56*i* and the incident wavelength *λ* is 632.8 nm. Larger diameter particles produce greater scattered light intensity with a more concentrated distribution in the front at small angles. With the change of particle size, the vertical and horizontal components of the scattered light show regular variation.

**Fig 5:**
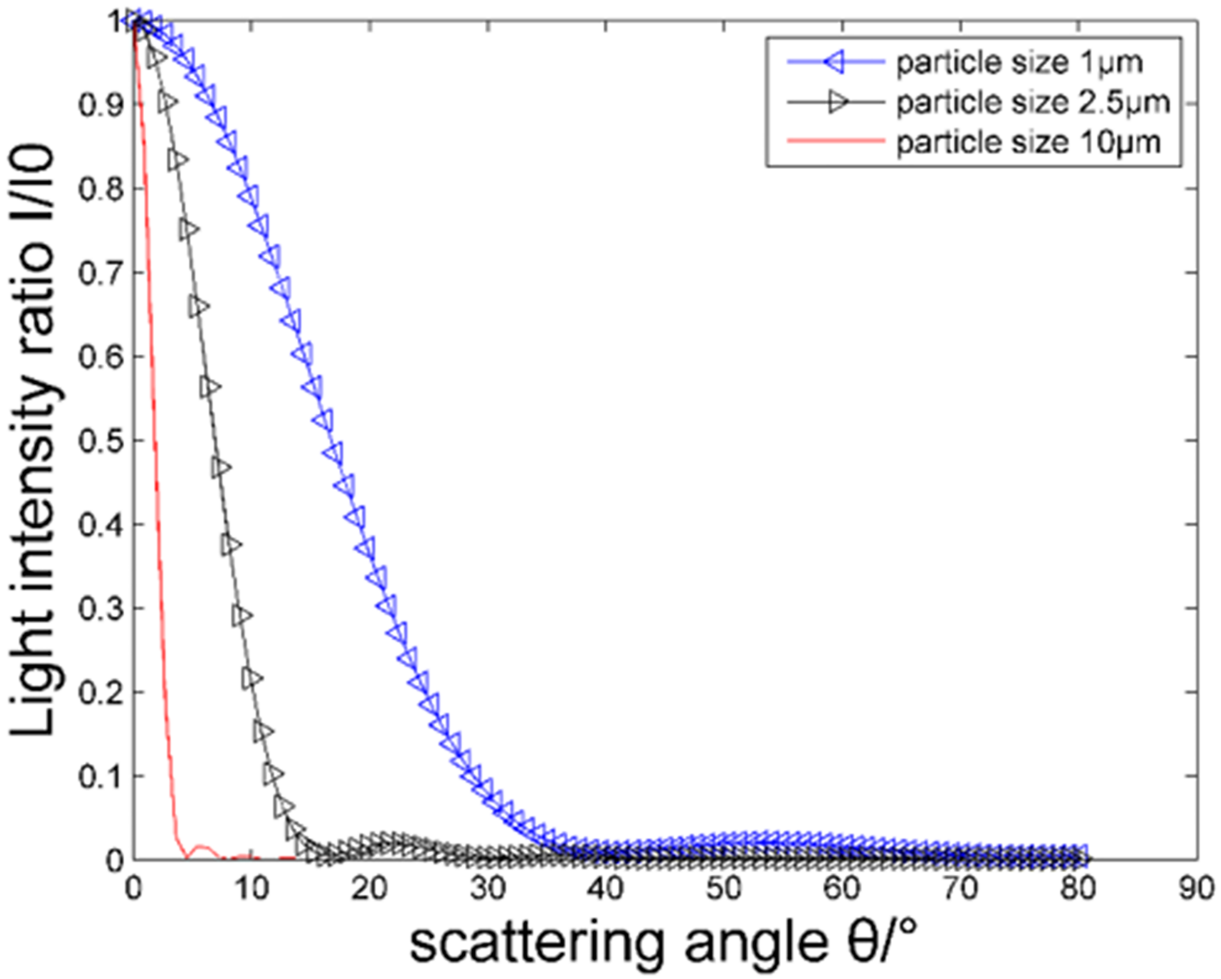
Angular distribution of the scattered light intensity for different particle sizes.

Fig 5 shows the effect of different particle sizes on the intensity distribution of the scattered light. The scattering caused by particle sizes larger than 10 μm is concentrated within the forward scattering angle of 5°, while that of particles between 2.5 μm and 10 μm is mainly concentrated at 8°, and that of particles between 1–2.5 μm is concentrated in the range of 10°. In this case, collecting the scattered light intensity from the front at small angles can be beneficial for obtaining information on 0.1–10 μm particles.

### Simulation verification

**Fig 6:**
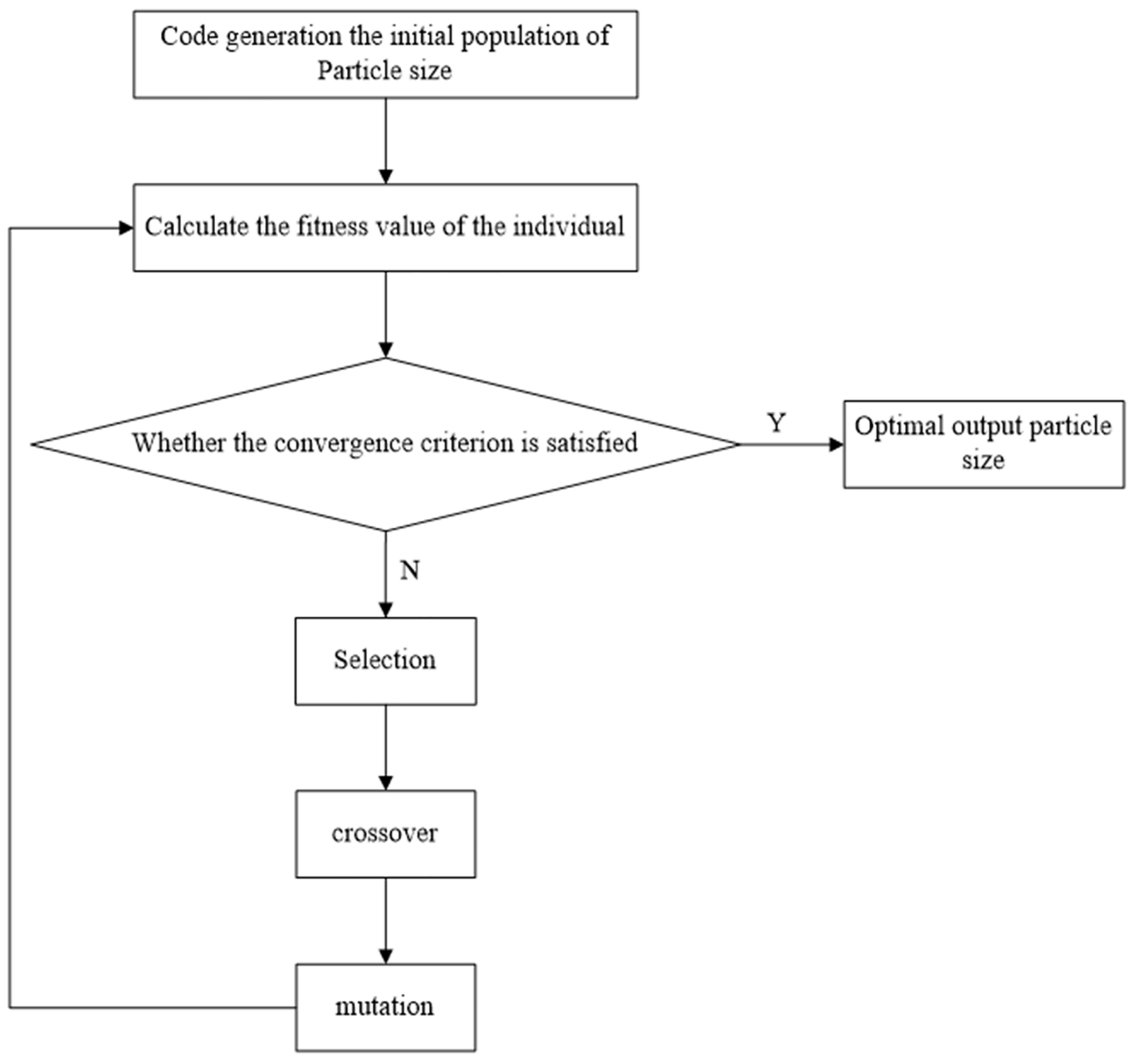
Inversion flow chart of the genetic algorithm.

According to Eq (1), the mass concentration of suspended particulate matter is the inverse process of determining the particle size. The inversion of particle size is essentially the solution process of the first Fredholm integral equation. The inversion flow chart was shown in Fig 6. Because the first kind of Fredholm integral equation is not a general solution, and the system of linear equations is ill conditioned by numerical or discretization under a large number of conditions, it is difficult to solve the linear equations using conventional methods. Therefore, the particle size distribution of suspended particulate matter has been solved by inversion of a genetic algorithm.

The Johnson-SB distribution is the commonly used two-parameter distribution in the inversion of particle size. The distribution is given as:

$$f\left(a\right)=\frac{1}{\sqrt{2\pi }}\frac{\sigma }{\alpha_{max}-\alpha_{min}}{\left[\frac{\alpha -\alpha_{min}}{\alpha_{max}-\alpha_{min}}\left(1-\frac{\alpha -\alpha_{min}}{\alpha_{max}-\alpha_{min}}\right)\right]}^{-1}exp\left\{-0.5{\left[\mu +\sigma Ln\left(\frac{\alpha -\alpha_{min}}{\alpha_{max}-\alpha }\right)\right]}^{2}\right\}$$ (11)

**Fig 7:**
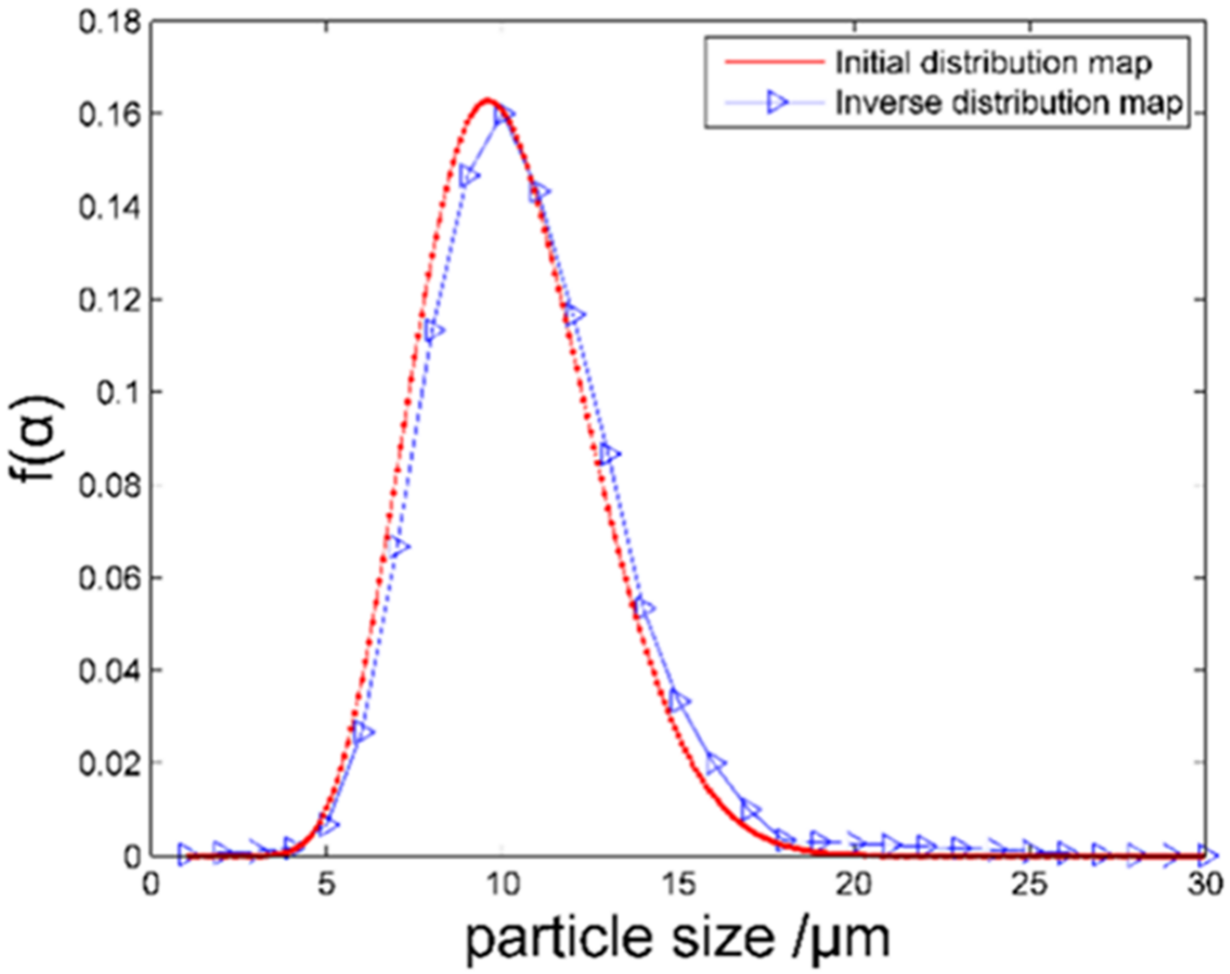
Inverse distribution map.

Fig 7 is the inverse distribution of particle size. If the initial distribution is Johnson-SB, we select σ = 2.5, *μ* = 2.0, and particle diameters in the range of 1–30 μm. With the above simulation standard, the incident light wavelength is selected to be 632.8 μm, the refractive index is 1.57–0.56*i*, and 15 sampling points are selected. The inversion results are in good agreement with the initial distribution, meeting theoretical requirements. Table 1 is the results of the inverse distribution of particle size.

**Table 1:** The inversion results.

Particle diameter/μm	1	2	3	4	5	6	7	8	9	10
**Original values**	0	2.050×10^−9^	2.082×10^−5^	1.150×10^−3^	1.033×10^−2^	3.804×10^−2^	8.320×10^−2^	1.294×10^−1^	1.581×10^−1^	1.607×10^−1^
**Inversion values**	0	1.927×10^−9^	2.001×10^−5^	1.098×10^−3^	0.518 ×10^−2^	2.782×10^−2^	7.320×10^−2^	1.097×10^−1^	1.479×10^−1^	1.603×10^−1^
**Particle diameter/μm**	11	12	13	14	15	16	17	18	19	20
**Original values**	1.408×10^−1^	1.087×10^−1^	7.494×10^−2^	4.645×10^−2^	2.593×10^−2^	1.301×10^−2^	5.824×10^−3^	2.302×10^−3^	7.897×10^−4^	2.296×10^−4^
**Inversion values**	1.455×10^−1^	1.163×10^−1^	8.143×10^−2^	5.547×10^−2^	2.913×10^−2^	1.879×10^−2^	6.136×10^−3^	3.149×10^−3^	7.905×10^−4^	2.311×10^−4^
**Particle diameter/μm**	21	22	23	24	25	26	27	28	29	30
**Original values**	5.471×10^−5^	1.018×10^−5^	1.377×10^−6^	1.214×10^−7^	5.860×10^−9^	1.136×10^−10^	4.812×10^−13^	1.035×10^−16^	6.922×10^−24^	0
**Inversion values**	5.562×10^−5^	1.123×10^−5^	1.462×10^−6^	1.358×10^−7^	5.946×10^−9^	1.241×10^−10^	4.971×10^−13^	1.123×10^−16^	0	0

### System hardware design

**Fig 8:**
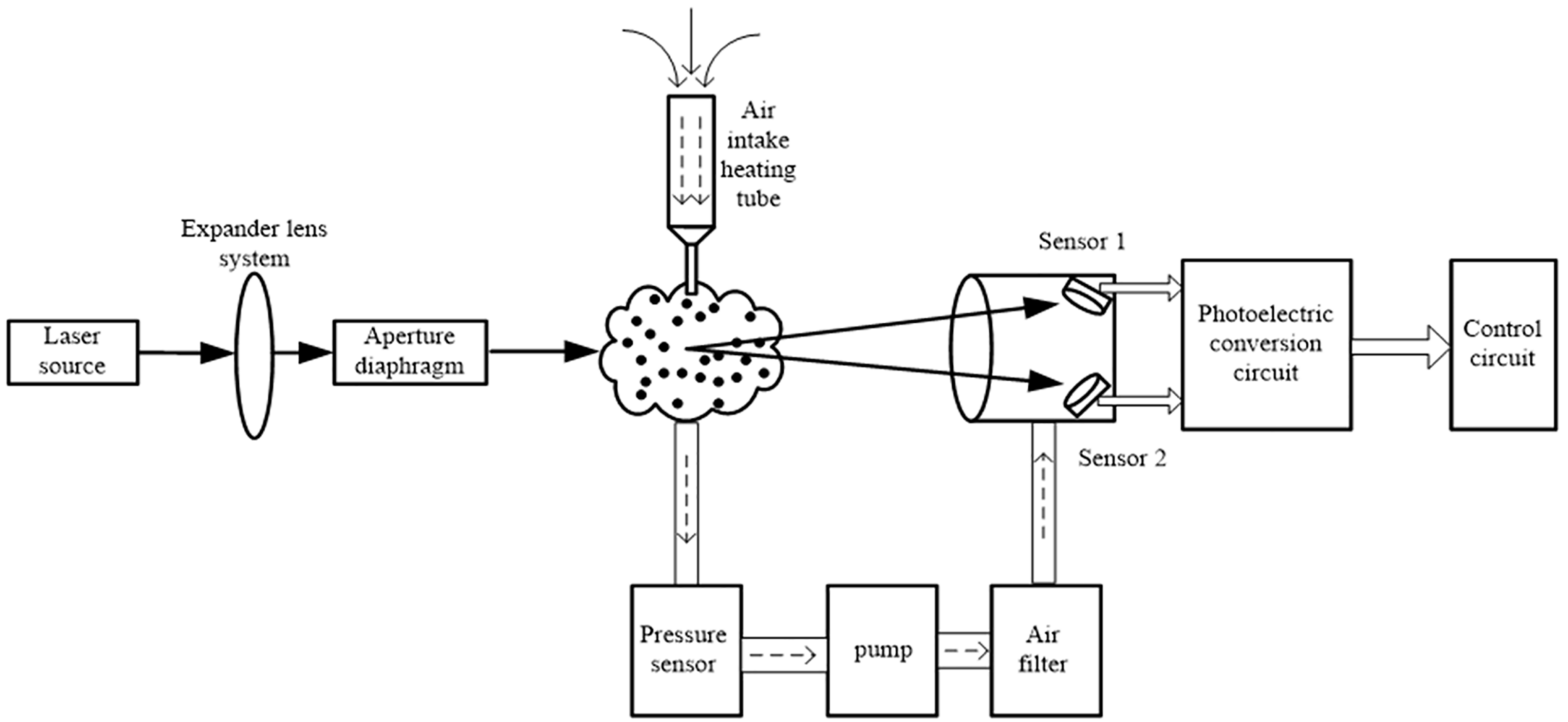
System light/gas path structure diagram.

The system hardware design is composed of five main parts: the optical path system, gas recovery system, photoelectric conversion subsystem, single chip microcomputer control unit, and other peripheral device units. The light path system consists of a semiconductor laser, a light source controller, and a lens group, forming an aperture. The photoelectric conversion subsystem is composed of two photoelectric detectors and a transmission circuit. Fig 8 shows the system light path structure.

The system light path structure primarily includes the functions of the incident laser and the acquisition of the scattered light. There are several components, including the laser, lens, aperture, heating pipe, gas chamber, pressure sensor, air pump, filter, two photoelectric sensors, sensor sending circuits, and main control circuit. The parameters of the laser are obtained according to the simulation results in the preceding part of this paper, using a HeNe laser with wavelength of 632.8 nm. The incident laser passes through a parallel beam expander by means of a lens group in order to meet the requirements of the range of incident light in the detection process. Stray light beams are passed through an aperture of the rear lens group in order to obtain a more ideal laser beam. When the light beam strikes dust in the air in the gas chamber, the scattering phenomenon occurs, and the scattered light irradiates two sensors at the back end of the air chamber that form a ±15° angle relative to the incident laser beam. The sensor (British LAB SMPX500 company’s SEME series) integrates a photodiode and an operational amplifier on the same chip, and a lens is provided on the surface of the package shell. In this way, the effect of leakage current and stray capacitance on measurement can be effectively reduced. The sensor receives a certain frequency of scattered light pulse signal. The amplitude of the signal and the size of the particle are related, and the frequency of the signal and the number of particles are related. Signals received by the sensor are transmitted to the instrument circuit system that performs the photoelectric conversion, dust concentration calculation, display, transmission, and other related work.

The main function of the pneumatic part of the system is to realize the function of sampling, filtering and flow measurement of environmental gases; it is mainly composed of an air-inlet pipe-heater, gas chamber, flow sensor, air pump, and the three-level filter element. The air intake heating pipe is located outside the instrument, heating the external air to reduce the influence of moisture in the air on the measurement accuracy. The air chamber is directly connected with the air-inlet heating pipe, providing the scattering environment for the incident light. The flow sensor using Honeywell company’s DC001NGC4 type ultralow-pressure sensor, with a range of 0~1″ H_2_O (0 to ~250 Pa), has an output signal of approximately 0.25 to 2.25 Vdc. Air pressure sensor output end is directly connected with the air pump, through gas pressure signal collected by front end pressure sensor control the inlet gas flow pump. Finally, take the air sampling which through the pump trough the two stage filter, get no dust air transmitted to the air chamber of the photoelectric sensor, in order to avoid the occurrence of complex scattering effect in the process of scattering light propagation.

**Fig 9:**
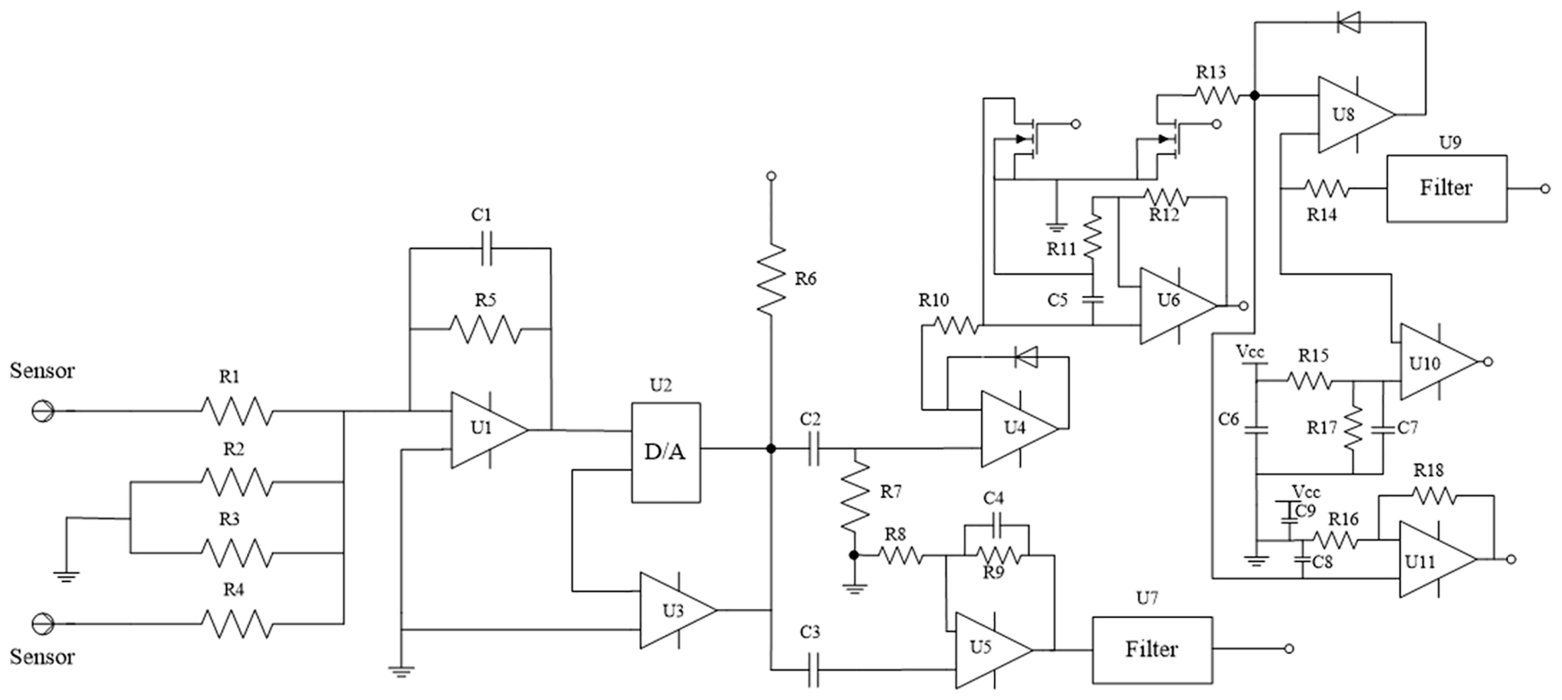
Schematic diagram of the photoelectric conversion circuit.

The circuitry of the instrument is composed of two parts. The first part is the photoelectric conversion unit in which the optical signal received by the sensor is transformed into a readable analog electrical signal, before being converted to a digital signal and transmitted to the main control circuit. The second part is the main control circuit of the system in which the calculation of the electric signal and dust concentration occurs along with the control of the laser light source, air pump flow control, dust concentration signal display, signal transmission, and keys operation, and other functions. The schematic diagram of the photoelectric conversion circuit is shown in Fig 9.

**Fig 10:**
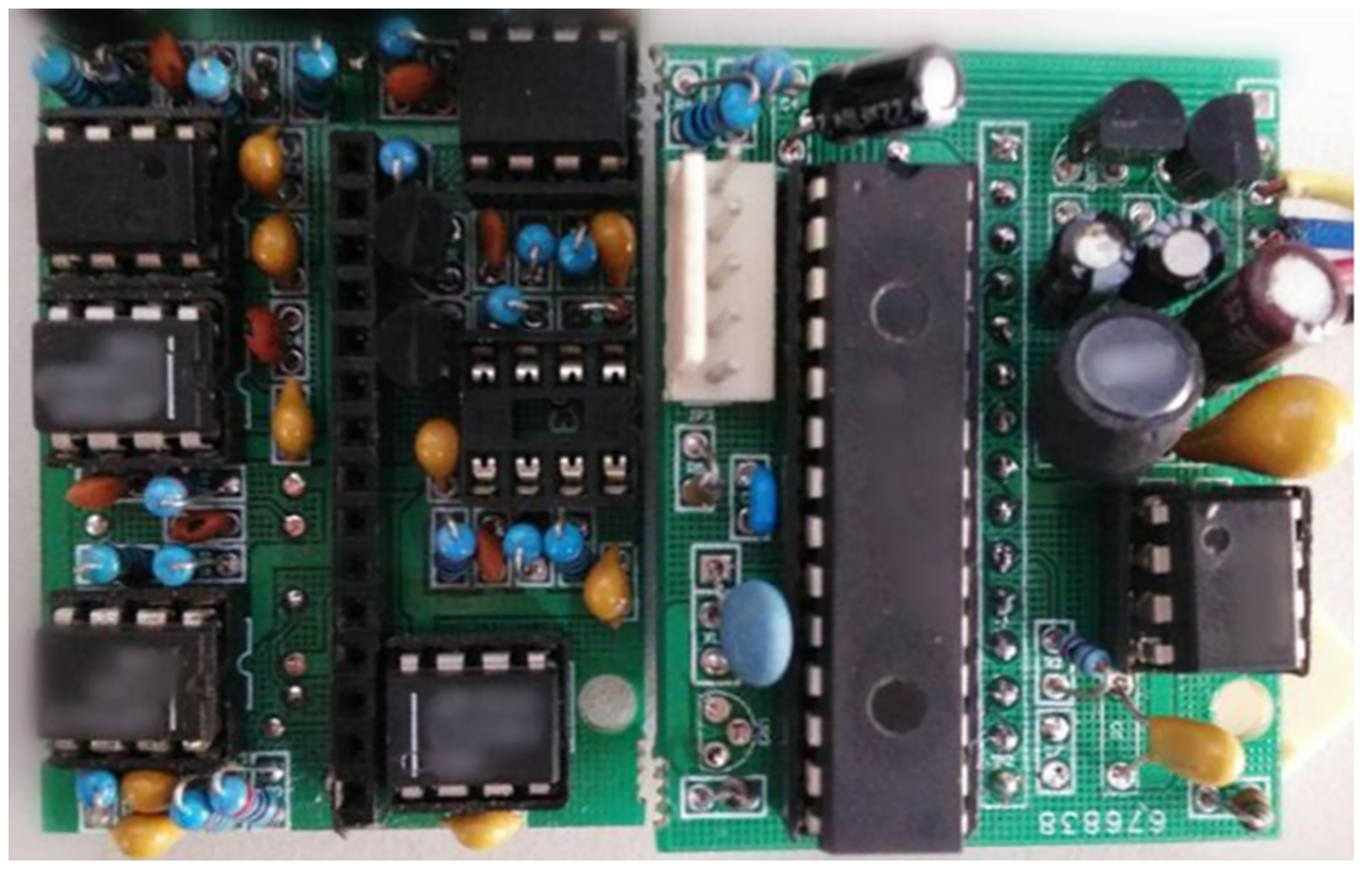
A photograph of the photoelectric conversion circuit.

In Fig 9, the first-level amplifier circuit amplifies the signal received by the sensor directly. Both sides of D/A chip U2 are connected across the feedback terminal operational amplifier U3, constituting a second stage programmable amplifier. The D/A chip is a type 8043, 12-bit digital-to-analog converter chip. We used the DAC8043 chip as a programmable resistance, rather than as a DAC. The T-type resistance network of the D/A chip as the feedback resistance of the two stage amplifier, taking feet 1 (Vref) and feet 2 (RFB) of chip 8043 are respectively across the input and output of the operational amplifier U3. To realize the programming of the feedback resistance of the two-stage amplifier, the output voltage of the two-stage amplifier is sampled by the signal chip microcomputer in the photoelectric conversion circuit according to the different output voltage regulating resistor network of D/A chip resistance. Here, U4 is an AC signal half-wave detector, whose is passed through the U6 amplifier and then through the low pass filter into the comparator. The output of the comparator is directly connected to an interruption of the signal chip microcomputer. Fig 10 shows a photograph of the photoelectric conversion circuit.

**Fig 11:**
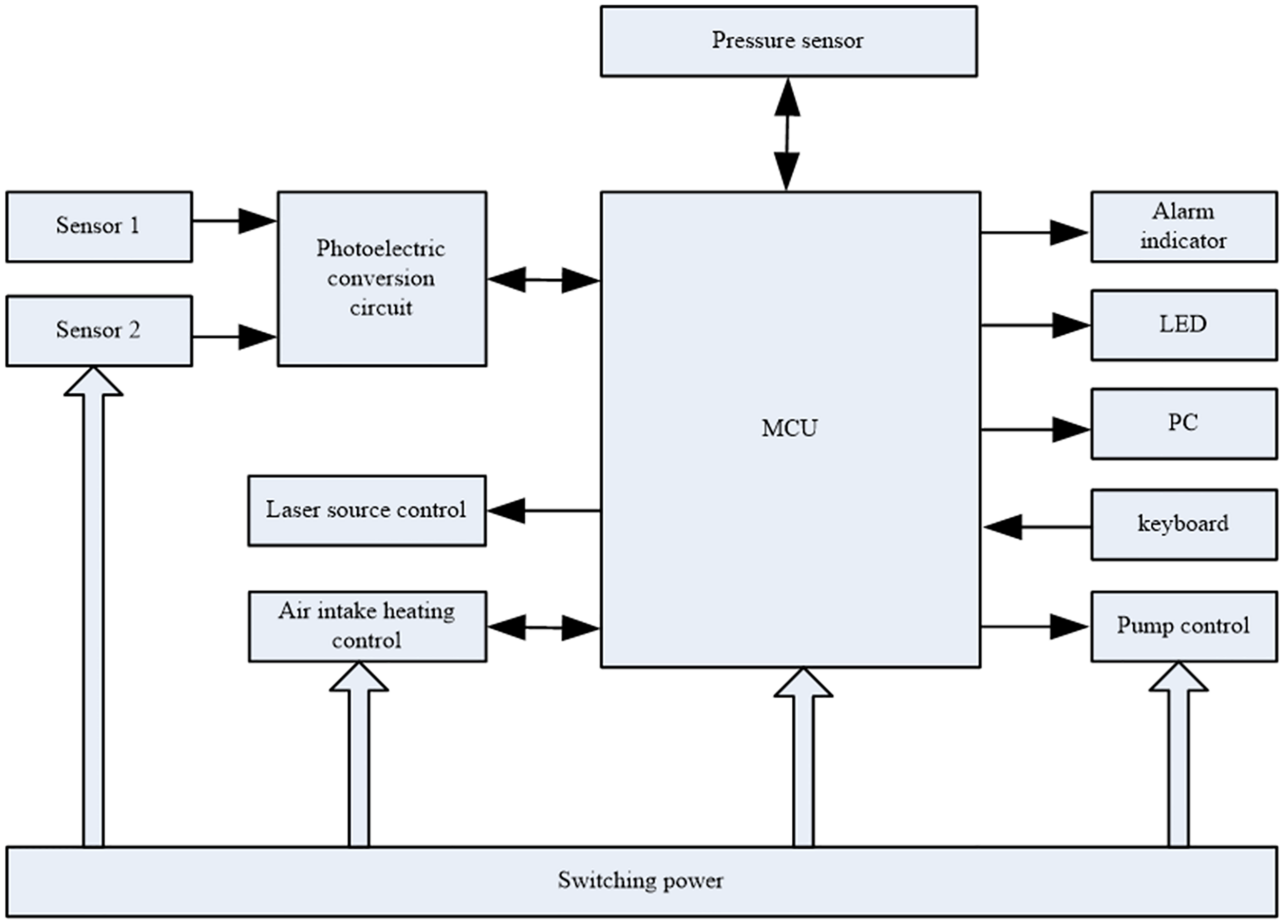
Detection system schematic.

**Fig 12:**
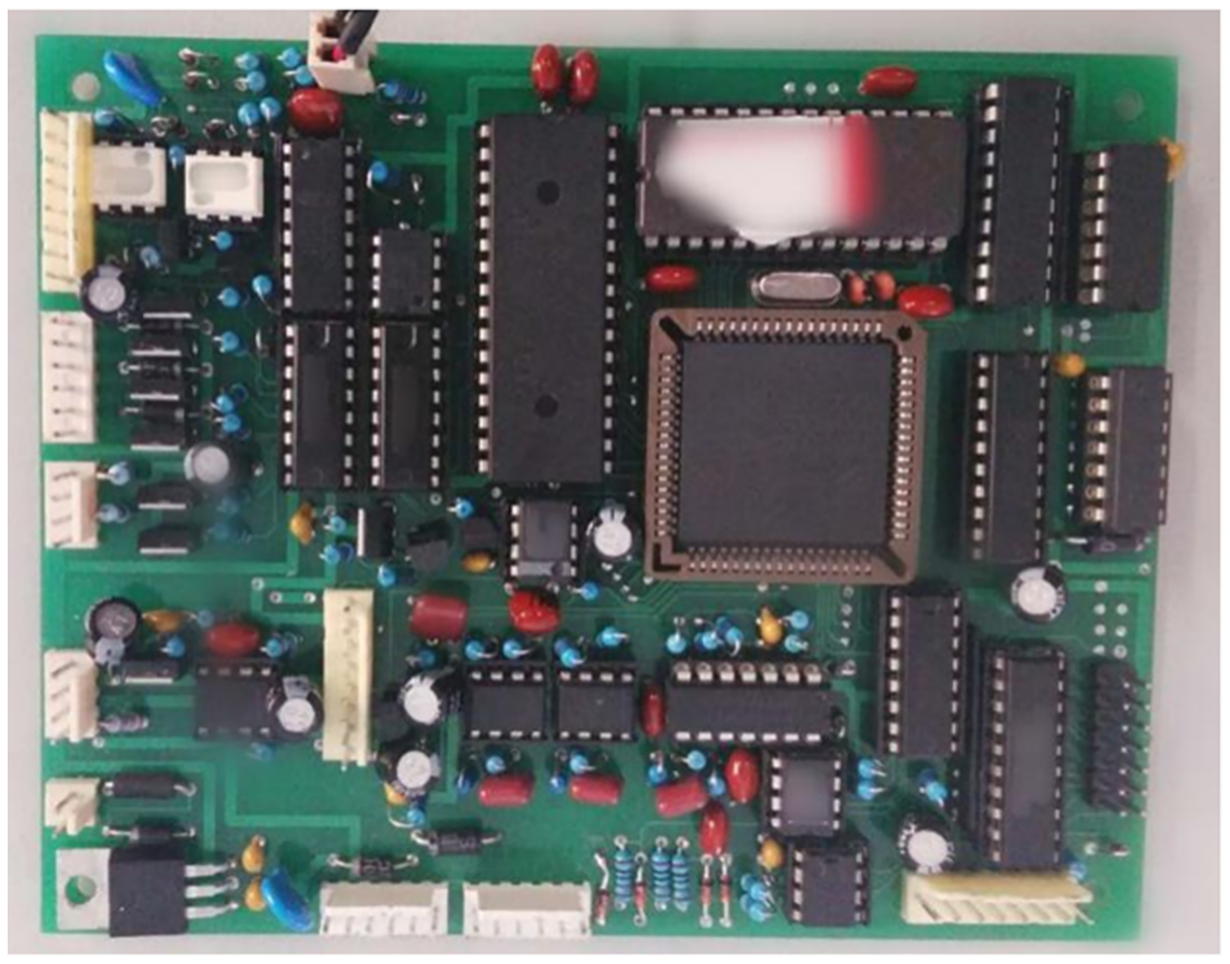
Circuit diagram of the main control circuit.

The block diagram and a photograph of the main control circuit are shown in Figs 11 and 12, respectively.

### Test result analysis

**Fig 13:**
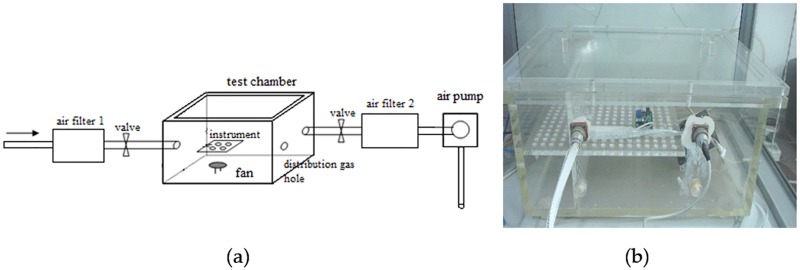
(a) Test box structure, (b) Photo of the test box.

The performance testing process of the instrument compares the results measured by the instrument with the weighing method. Before the experiment, the tightness of the organic glass test chamber and the sealed gas path are verified, and the instrument is placed inside the box body. At this point, air filter 1 (PM2.5) is installed. After the start of the experiment, the pump is opened to filter the air inside the test box, removing air particulate matter from the test chamber; then the pump is closed and the PM2.5 filter is replaced by the PM10 filter in air filter 1 position. Air filter 2 (PM2.5) is installed, and the pump is opened. When the instrument shows a stable number, the number is read, the pump is closed, and air filter 2 is removed and weighed. Fig 13 shows the test box structure and a photograph.

**Fig 14:**
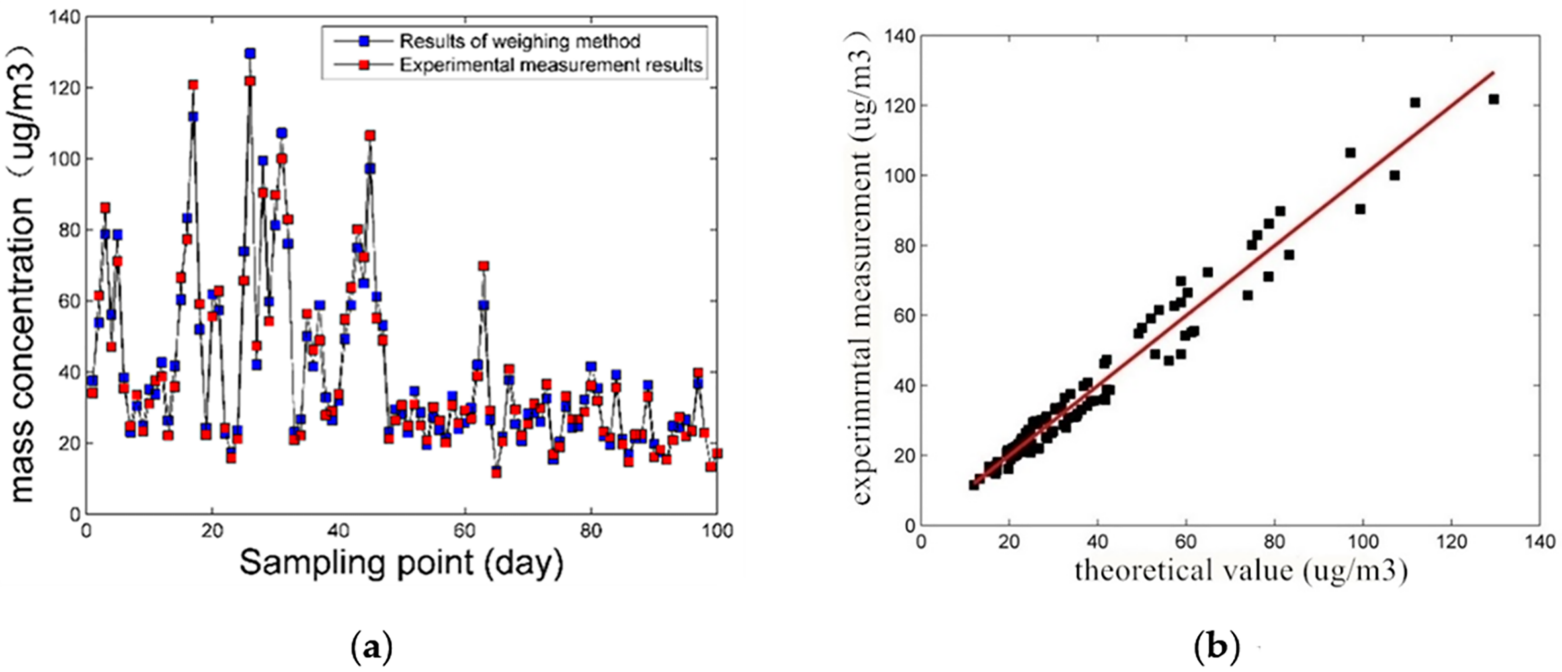
The relationship curve between light-scattering experiment measured data and the weighing experiment data of PM2.5.

Fig 14(a) and 14(b) are the relationship curve between light-scattering experiment measured data and the weighing experiment data of PM2.5. As shown in Fig 14(b), the two sets of data track each other very closely, and the correlation coefficient is 0.982.

**Fig 15:**
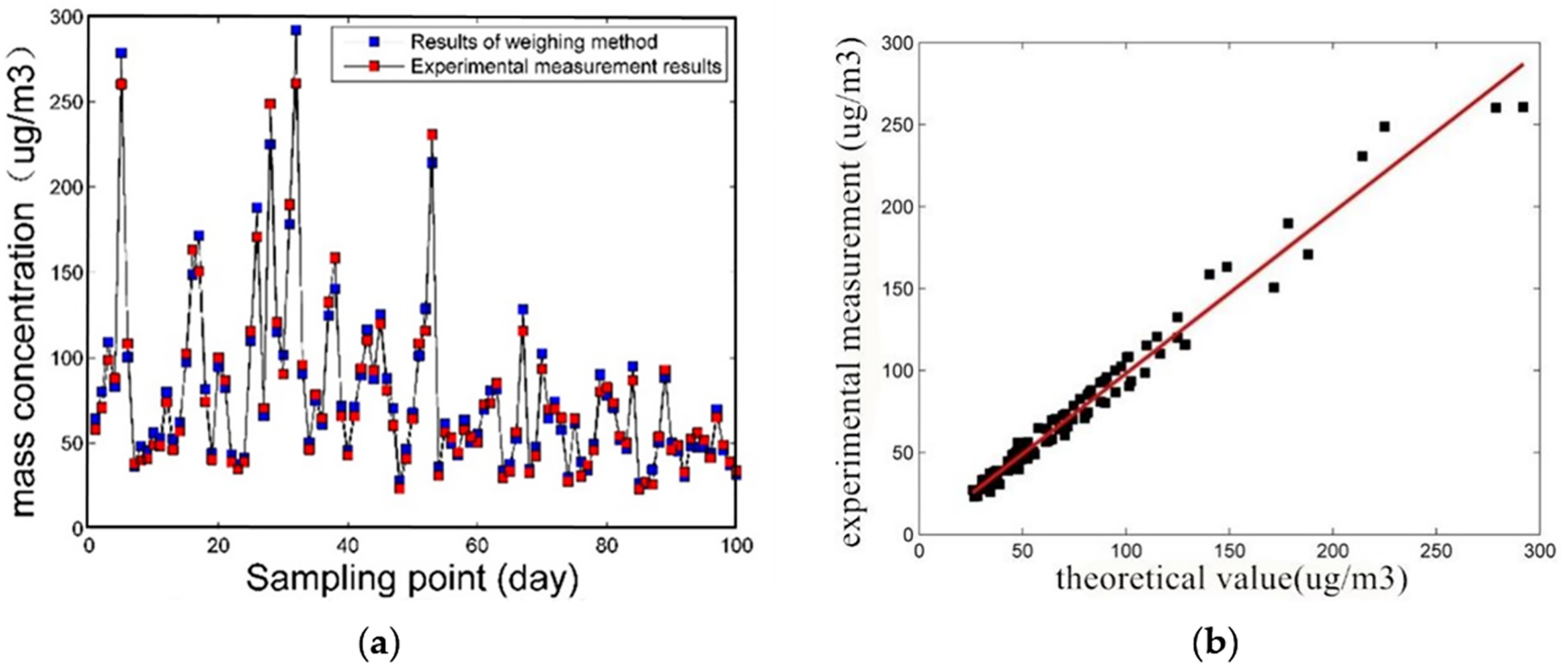
The relationship curve between light-scattering experiment measured data and the weighing experiment data of PM10.

Fig 15(a) and 15(b) are the relationship curve between light-scattering experiment measured data and the weighing experiment data of PM10. From Fig 15(b) can be seen, correlation coefficient is 0.987, the results show that the system has high measurement precision.

## Conclusion

Based on the principle of laser scattering and using Mie scattering theory to simulate the light scattering intensity of suspended particles with MATLAB, the effects of the incident wavelength λ, refractive index *m* (real part and imaginary part), and the particle size on the distribution of scattered light intensity were analyzed. The designed dust-concentration detection system can realize real-time on-line detection of PM 1, PM 2.5, PM 10, and Total Suspended Particulates (TSP) concentrations in air. Through field experiments, it was demonstrated that the correlation coefficient between the light-scattering experiment measured data and the weighing experiment data of particle concentration is less than 0.982 of PM2.5 and 0.987 of PM10. Thus, the system designed in this paper has good accuracy, meeting the requirements for measuring dust concentration with different particle sizes.

## Supporting information

S1 File Empirical data. (ZIP)
